# Consequences of increased terrestrial dissolved organic matter and temperature on bacterioplankton community composition during a Baltic Sea mesocosm experiment

**DOI:** 10.1007/s13280-015-0659-3

**Published:** 2015-05-28

**Authors:** Markus V. Lindh, Robert Lefébure, Rickard Degerman, Daniel Lundin, Agneta Andersson, Jarone Pinhassi

**Affiliations:** Centre for Ecology and Evolution in Microbial model Systems - EEMiS, Linnaeus University, 391 82 Kalmar, Sweden; Department of Ecology and Environmental Science, Umeå University, 901 87 Umeå, Sweden; Marine Stewardship Council, 1 Snow Hill, London, EC1A 2DH UK

**Keywords:** Terrestrial dissolved organic matter, Temperature, Climate change, Marine bacteria, Bacterial diversity

## Abstract

Predicted increases in runoff of terrestrial dissolved organic matter (DOM) and sea surface temperatures implicate substantial changes in energy fluxes of coastal marine ecosystems. Despite marine bacteria being critical drivers of marine carbon cycling, knowledge of compositional responses within bacterioplankton communities to such disturbances is strongly limited. Using 16S rRNA gene pyrosequencing, we examined bacterioplankton population dynamics in Baltic Sea mesocosms with treatments combining terrestrial DOM enrichment and increased temperature. Among the 200 most abundant taxa, 62 % either increased or decreased in relative abundance under changed environmental conditions. For example, SAR11 and SAR86 populations proliferated in combined increased terrestrial DOM/temperature mesocosms, while the hgcI and CL500-29 clades (Actinobacteria) decreased in the same mesocosms. Bacteroidetes increased in both control mesocosms and in the combined increased terrestrial DOM/temperature mesocosms. These results indicate considerable and differential responses among distinct bacterial populations to combined climate change effects, emphasizing the potential of such effects to induce shifts in ecosystem function and carbon cycling in the future Baltic Sea.

## Introduction

Predicted climate change, resulting in effects such as increased sea surface temperatures and precipitation, threatens the structure and function of marine communities in many regions of the oceans, including the Baltic Sea and coastal waters in general (Meier 2006; IPCC 2013). Although marine bacteria play an essential role in driving biogeochemical cycling of, e.g., carbon, knowledge on how these microorganisms will be affected by anthropogenic impacts is scarce. Still, increased temperature is known to affect growth and drive compositional shifts in marine microbial communities (Müren et al. 2005; von Scheibner et al. 2014). In addition to temperature changes, the future Baltic Sea is predicted to experience an increase in terrestrial nutrient runoff from rivers, including terrestrial dissolved organic matter (tDOM), due to increased annual levels of precipitation (Meier 2006). Such tDOM will include humic substances comprised of low- and high molecular weight compounds like fulvic acids and lignin (Rocker et al. 2012). Increases in terrigenous organic matter could induce changes in food web dynamics and energy flows in the system (Sandberg et al. 2004; Wikner and Andersson 2012). Although the importance of DOM composition in structuring bacterioplankton communities is relatively well established (where phytoplankton-derived compounds are most studied, e.g., Gomez-Consarnau et al. 2012; Teeling et al. 2012; Dinasquet et al. 2013), few studies have considered the importance of tDOM input (mainly humic substances derived from river runoff) in driving bacterioplankton compositional shifts in marine systems (but see Kisand et al. 2002; Rochelle-Newall et al. 2004; Kisand et al. 2008; Teira et al. 2009; Grubisic et al. 2012; Rocker et al. 2012). However, the impact of humic matter on bacterioplankton composition has been extensively investigated in limnic systems (e.g., Lindström 2000; Eiler et al. 2003; Kritzberg et al. 2006). Typically, there are few general patterns among bacterioplankton at the phyla/class level in these studies and only a handful have analyzed the distribution of specific bacterial populations. Nevertheless, Bacteroidetes, Gammaproteobacteria, and Betaproteobacteria seem to be prevalent in relation to environmental conditions with high tDOM (Kisand et al. 2002; Eiler et al. 2003; Teira et al. 2009). Collectively these studies show that growth and community structure of bacterioplankton much depend on the ability of bacteria to degrade and utilize tDOM. Considering the importance of DOM in shaping bacterioplankton community structure, surprisingly few studies have investigated the potential effects of climate change-induced increases in tDOM on bacterioplankton community composition.

In addition to changes in single environmental variables, simultaneous shifts in both DOM composition and increased sea surface temperatures may have even larger consequences for bacterioplankton. In the equatorial Pacific Ocean and the Western Arctic Ocean, autochthonous dissolved organic carbon (DOC) and increased temperature caused synergistic effects on bacterial growth (Kirchman and Rich 1997). In the northern Baltic Sea, increased temperature regulated bacterioplankton composition to a small extent, while high terrestrial DOM input was important in determining community structure (Degerman et al. 2013). However, in that study, the potential combined effect of temperature and terrestrial DOM was not investigated. These findings highlight the potential importance of climate change effects in shaping the structure and function of marine ecosystems in general and also for bacterioplankton dynamics. Still, the potential effects of increased tDOM concentrations and temperature on bacterial community structure and the relative abundance of individual bacterial populations remain largely unknown.

It is generally recognized that bacterioplankton populations (frequently defined as operational taxonomic units—OTUs) have a remarkable potential in responding to environmental disturbances (Langenheder et al. 2005; Allison and Martiny 2008; Comte and Del Giorgio 2011; Sjöstedt et al. 2012). However, the ecological significance of the adaptability of bacterioplankton populations, or their physiological plasticity, in responding to synergistic environmental disturbances as highlighted above, is poorly understood. In responding to climate change-induced environmental perturbations, bacterial populations can either be sensitive (i.e., decrease in relative abundance), resistant (i.e., maintain relative abundance), or responsive (i.e., increase in relative abundance) (Allison and Martiny 2008). In addition, how individual bacterial populations differ in their response to environmental disturbance will likely have implications for a number of bulk community properties (e.g., bacterial production) that heavily influence ecosystem functioning by changing the flow of carbon (Bell et al. 2005; Comte and Del Giorgio 2011). Further knowledge on the details of gains and losses of bacterial populations in response to environmental changes, such as increased tDOM loading and temperature, would be desirable. This would be critically important for disentangling the effects of climate change on bacterioplankton assemblages and their ecosystem function in the future Baltic Sea.

Lefébure et al. (2013) showed substantial synergistic effects of increased tDOM and temperature on different trophic levels in the Baltic Sea. For example, both fish production and food web efficiency were higher in mesocosms with manipulated environmental conditions compared to controls. Using samples from this experiment, we aimed at investigating the potential future climatic effects of changes in temperature and tDOM on bacterioplankton community composition, specifically the dynamics of individual OTUs. We used 16S rRNA gene tag pyrosequencing analysis on samples collected from mesocosms exposed to combined increases in temperature and tDOM concentration as compared to controls (each in triplicates). In addition to detecting overall changes in bacterial community composition between control mesocosms and mesocosms with increased tDOM and temperatures, we hypothesized the experiment would allow identifying specific bacterial populations (OTUs) that are particularly sensitive, resistant, or responsive to the environmental forcing.

## Materials and methods

### Experimental setup

The mesocosm experiment, to simulate the effects of increased river-bound input of tDOM and increased surface seawater temperatures (tDOM_H_ + T) into the northern Baltic Sea, was performed at Umeå Marine Research Centre, Sweden. Each mesocosm contained 2000 l unfiltered water collected in the Bothnian Sea in October 2010 (6 °C, salinity 5), (63°34′N, 19°54′E). We used four experimental treatments with three replicates each. In this study, our focus is on two of these treatments, tDOM addition and temperature increase vs. control conditions. tDOM was added to increase DOC concentration from 4.5 mg l^−1^ in control mesocosms to 6 mg C l^−1^ in tDOM_H_ + T mesocosms. Temperature was initially raised to 15 °C in all mesocosms (“stabilization phase” for 18 days) to ensure equal starting point and then by another 4 °C in the tDOM_H_ + T mesocosms to 19 °C during 35 days. Mesocosms were kept at a constant temperature (±0.5 °C). For detailed descriptions of the experimental setup and sampling of biotic and abiotic environmental parameters, see (Lefébure et al. 2013).

### Collection and extraction of community DNA

Biomass for DNA extraction was collected at the stabilization phase (i.e., prior to the experiment start) and then at the start (day 0), middle (day 14), and end of the experimental phase (day 28 and 35). Samples of 0.5–1.0 l were filtered onto 0.2 µm pore size, 47-mm diameter Supor filters (PALL Life Sciences). The filters were immediately frozen at −80 °C in 1.8 ml TE buffer (10 mM Tris, 1 mM EDTA, pH 8.0) until further processing. DNA extraction was performed according to the phenol chloroform extraction protocol in Riemann et al. (2000). Bacterial 16S rRNA genes were amplified with bacterial primers 341F and 805R, containing adaptor and barcode following the protocol of Herlemann et al. (2011). The resulting purified barcoded amplicons were normalized in equimolar amounts and sequenced on a Roche GS-FLX 454 automated pyrosequencer (Roche Applied Science, Branford, CT, USA) at Science for Life Laboratory, Stockholm, Sweden.

### Sequence processing and analysis

Raw sequence data generated from 454 pyrosequencing were processed following the bioinformatical pipeline described in Lindh et al. (2015). The 454 run resulted in 80 000 reads. After denoising and chimera removal, samples contained on average 2763 (±597) sequence reads for each sample. The final OTU table, including chloroplast sequences, consisted of 1688 OTUs (excluding singletons). DNA sequences have been deposited in the National Center for Biotechnology Information (NCBI) Sequence Read Archive under accession number SRP036629.

### Statistical analyses and graphical outputs

All graphical outputs were performed in R 3.0.2, and statistical tests were made using the package Vegan (Oksanen et al. 2010). Clusters in nMDS analysis were drawn based on visual difference between samples.

## Results

### Microbial community composition

**Fig. 1:**
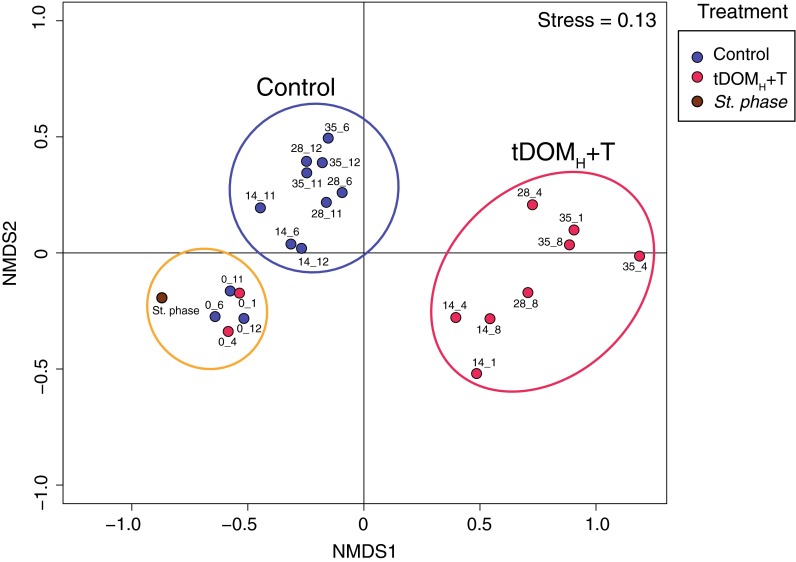
nMDS analysis based on 454 pyrosequencing data of control mesocosms and mesocosms of increased terrestrial dissolved matter with increased temperature (tDOM_H_ + T). *Circles* denote distinct clusters based on visual grouping of samples. Each sample is designated by experimental day and mesocosm number (6, 11 and 12 = control, 1, 4 and 8 = tDOM_H_ + T). *St. phase* stabilization phase prior to the experiment

Analysis of pyrosequencing data on microbial community composition by non-metric multidimensional scaling (nMDS) showed that the initial samples on day 0 clustered together and close to the sample from the end of the stabilization phase (Fig. 1). A distinct grouping of samples distinguishing control mesocosms from mesocosms with increased terrestrial humic DOM and temperature (tDOM_H_ + T) was observed already in the middle of the experiment (day 14). This pattern was consistent among replicates for control and tDOM_H_ + T mesocosms, and was maintained until the end of the experiments (days 28 and 35) (Fig. 1). The microbial community composition in the control and tDOM_H_ + T mesocosms was significantly different (PerMANOVA, *p* = 0.01, n = 23).

### Population dynamics

**Fig. 2:**
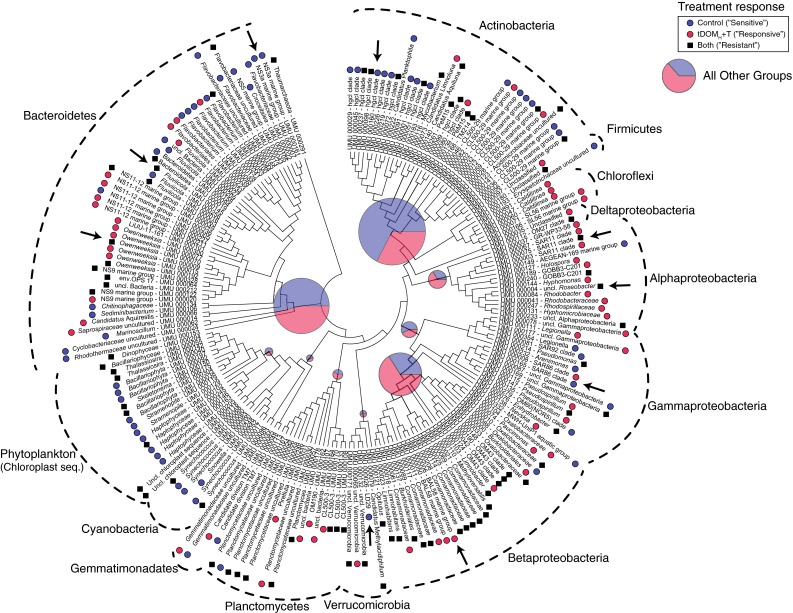
Maximum Likelihood (ML)-based phylogenetic tree of 16S rRNA gene sequences obtained from 454 data. ML tree was calculated from nearest neighbor interchange (NNI). Pie charts indicate average relative abundance for each major bacterial group (including all OTUs) in control and tDOM_H_ + T mesocosms. The size of each pie chart is proportional to total average relative abundance. Differential response in relative abundance of the top 200 most abundant OTUs is indicated by *blue filled circles* for OTUs responding in control mesocosms (“sensitive”), *pink filled circles* for OTUs responding in tDOM_H_ + T mesocosms (“responsive”), and *black filled squares* for OTUs with unchanged response (“resistant”). *Arrows* denote particularly important OTUs mentioned in discussion. *Scale bar* represents 0.1 nucleotide substitutions per site

Differences between control and tDOM_H_ + T mesocosms in terms of community composition resulted mainly from the gradual increase and decrease in the relative abundance of different bacterial populations (defined as OTUs, at 97 % of sequence identity of the 16S rRNA gene) and much less from the presence/absence of specific OTUs. Therefore, we investigated the distribution patterns of the 200 most abundant OTUs over the entire experiment (accounting for 88 % of total reads), summarized in Fig. 2. Further details on the 20 most abundant OTUs are summarized in Table 1. Among the top 200 OTUs, 30 % were more abundant in tDOM_H_ + T mesocosms and 32 % were less abundant in tDOM_H_ + T mesocosms compared to control mesocosms. A large proportion of the OTUs, 38 %, were resistant to manipulation, i.e., responded similarly in tDOM_H_ + T mesocosms and the controls in terms of increasing or maintaining the relative abundance. At the phyla/class level, several actinobacterial OTUs were less abundant in tDOM_H_ + T mesocosms as in the controls, indicating that they were sensitive to this change in growth conditions (Fig. 2). Bacteroidetes OTUs were more diverse in their response to tDOM_H_ + T, where some OTUs preferred control conditions, while others were predominant in tDOM_H_ + T mesocosms (Fig. 2). Still, other OTUs within Bacteroidetes were maintained or increased in relative abundance in both control and tDOM_H_ + T mesocosms. Similarly, Gammaproteobacteria contained several OTUs responding either mostly to control or tDOM_H_ + T mesocosms and some that were unchanged or increased equally between these conditions. Moreover, a large number of Betaproteobacteria were found in high relative abundance in our study and were also quite variable in their response, but a majority was more abundant in tDOM_H_ + T mesocosms, indicating that they were responsive. Several Alphaproteobacteria OTUs increased in tDOM_H_ + T mesocosms or were unchanged between controls and simulated climate change. Cyanobacteria and phytoplankton (chloroplast sequences) displayed higher relative abundance in control compared to tDOM_H_ + T mesocosms (Fig. 2).

**Table 1:** Response of the top 20 most abundant OTUs over the experiment. Phyla/Class is abbreviated; Actino.—Actinobacteria, Alpha—Alphaproteobacteria, Bact.—Bacteroidetes, Beta—Betaproteobacteria, Gamma—Gammaproteobacteria. Sequence annotation was performed with SINA/SILVA and also using manual BLAST showing the Accession number of the closest relative found in genbank and 16S rRNA gene identity in percent. Asterisks (*) indicate relation to phylotypes previously found in the Baltic Sea and (§) indicates observations in past nutrient amendment experiments. Average relative abundance and maximum relative abundance (in parenthesis) during the experiment are given in percent. We define abundant populations as having >1 % in relative abundance and rare populations as having <0.1 %. The detection limit for this study is around 0.03 %, based on the sequencing depth. Magnitude of response in control and TDOM_H_ + T mesocosms is indicated with + (present) or − (absent). The level of response is indicated by the number of +, which is relative for each OTU

OTU	Taxa (SINA/SILVA)	Acc. # (GenBank)	Phyla/class	Rel. abund. (All)	Rel. abund. (control)	Rel. abund. (tDOM_H_ + T)	Control (“sensitive”)	tDOM + T (“responsive”)
UMU_000001	hgcI clade	FR647689.1 [100 %]*	Actino.	7.8 (20.4)	11.2 (20.4)	3.8 (7.3)	+ + +	+
UMU_000002	CL500-29	HQ836442.1 [100 %]*§	Actino.	6.2 (11.9)	8.8 (11.9)	3.2 (10.2)	+ + +	+
UMU_000012	CL500-29	AB831248.1 [99 %]§	Actino.	0.9 (2.5)	1.2 (2.5)	0.5 (2.0)	+ +	+
UMU_000028	CL500-29	DQ270295.1 [100 %]*	Actino.	1.0 (2.5)	0.9 (1.7)	1.1 (2.5)	+	+ +
UMU_000029	hgcI clade	AB831241.1 [100 %]§	Actino.	1.0 (3.2)	1.6 (3.2)	0.2 (0.8)	+ +	+
UMU_000051	hgcI clade	AB831253.1 [100 %]§	Actino.	1.0 (1.7)	1.2 (1.7)	0.8 (1.2)	+ +	+ +
UMU_000003	SAR11	JQ974826.1 [100 %]*	Alpha	2.9 (13.8)	1.6 (6.5)	4.6 (13.8)	+	+ + +
UMU_000004	uncl. *Roseobacter*	FR647982.1 [100 %]*	Alpha	10.2 (15.9)	10.5 (15.9)	10 (14.7)	+ + +	+ + +
UMU_000005	NS11-12	FR647978.1 [100 %]*	Bact.	1.7 (4.5)	2.5 (4.5)	0.7 (1.8)	+ +	+
UMU_000007	NS3a	KC899250.1 [100 %]	Bact.	2.0 (6.8)	3.3 (6.8)	0.5 (2.2)	+ + +	+
UMU_000009	*Owenweeksia*	FJ744887.1 [100 %]	Bact.	1.2 (5.2)	0.8 (1.8)	1.6 (5.2)	+	+ + +
UMU_000013	*Flavobacteriaceae*	DQ189595.1 [99 %]§	Bact.	1.4 (5.6)	1.7 (5.6)	1.0 (2.4)	+ +	+ +
UMU_000015	*Owenweeksia*	EU878165.1 [100 %]§	Bact.	1.2 (5.3)	0.8 (1.3)	1.8 (5.3)	+	+ + +
UMU_000016	*Fluviicola*	FR691964.1 [100 %]§	Bact.	1.4 (3.4)	1.8 (3.4)	0.9 (3.2)	+ +	+
UMU_000024	*Cyclobacteriaceae*	HQ836440.1 [100 %]*§	Bact.	1.3 (3.2)	1.7 (3.2)	0.8 (1.7)	+ +	+
UMU_000000	*Burkholderia*	JN371511.1 [100 %]	Beta	7.7 (41.5)	3.2 (6.7)	13.1 (41.5)	+	+ + +
UMU_000006	BAL58	HQ836424.1 [100 %]*§	Beta	1.6 (4.1)	1.3 (4.1)	2.9 (3.5)	+	+ +
UMU_000010	*Oxalobacteraceae*	FJ828452.1 [100 %]§	Beta	1.6 (5.9)	1.8 (5.9)	1.3 (4.8)	+ +	+ +
UMU_000011	*Comamonadaceae*	EU167462.1 [100 %]§	Beta	1.0 (5.3)	0.0 (0.0)	2.2 (5.3)	−	+ +
UMU_000008	SAR86	FR647697.1 [100 %]*	Gamma	1.7 (9.0)	0.5 (2.3)	2.8 (9.0)	+	+ + +

Within Actinobacteria, most members of the hgcI clade (for example UMU_000001, UMU_000029) were predominant in control mesocosms (Fig. 2; Table 1). Similarly, most Actinobacteria members of the CL500-29 clade were also more abundant in control compared to tDOM_H_ + T mesocosms (for example, UMU_000002, UMU_000012). However, one CL500-29 clade OTU (UMU_000082) responded by increasing more in tDOM_H_ + T mesocosms than in controls (Fig. 2).

**Table 2:** Recruitment of rare OTUs. Examples of OTUs undetected during stabilization phase and day 0 of the experiment but later detected in tDOM_H_ + T mesocosms. Phyla/Class is abbreviated; Bact.—Bacteroidetes, Beta—Betaproteobacteria, Delta—Deltaproteobacteria, and Chlor.—Chloroflexi. Average relative abundance and maximum relative abundance (in parenthesis) during the experiment are given in percent

OTU	Taxa	Taxon	Rel. abund.
UMU_000020	NS9 marine group	Bact.	1.5 (3.9)
UMU_000027	*Fluviicola*	Bact.	1.0 (2.7)
UMU_000011	*Comamonadaceae*	Beta	2.2 (5.3)
UMU_000037	*Desulfuromonadales*	Delta	1.0 (6.7)
UMU_000060	*Caldilinea*	Chlor.	0.5 (2.1)

Among Bacteroidetes, most *Owenweeksia*-related OTUs (UMU_000009, UMU_000015, UMU_000077, UMU_000300) increased in abundance in tDOM_H_ + T mesocosms, whereas NS3a clade OTUs were abundant mainly in control mesocosms (UMU_000007, UMU_000161). Interestingly, one OTU (UMU_000019) related to *Fluviicola* was abundant in tDOM_H_ + T mesocosms on day 14 but decreased toward the end of the experiment, while the same OTU in the control mesocosms was low in abundance on day 14 but increased toward the end (Table 2). In addition, another *Fluviicola* relative (UMU_000027) was not detected in the beginning of the experiment but later increased substantially in tDOM_H_ + T mesocosms (Table 2). Similarly, an NS9 relative (UMU_000020) was also recruited from being undetected to become abundant in tDOM_H_ + T mesocosms (Table 2). Concomitantly, a member of the numerically abundant SAR86 clade was predominantly abundant in control mesocosms (UMU_000165) and another SAR86 OTU was higher, at around 2.8 % in average relative abundance (reaching up to 10 %), in tDOM_H_ + T mesocosms (UMU 000008) (Fig. 2; Table 1).

Most *Comamonadaceae* (Betaproteobacteria) were abundant in the tDOMH + T mesocosms on day 14, but then decreased substantially toward the end of the experiment. However, three OTUs (UMU_000006, UMU_000050 and UMU_000011), closely related to BAL58 marine group, further increased in relative abundance in tDOMH + T mesocosms after day 14 (Fig. 2; Table 1). Another Betaproteobacteria OTU, related to *Burkholderiales* (UMU_000000), responded substantially in tDOM_H_ + T mesocosms where it reached over 40 % of relative abundance (Fig. 2; Table 1). Interestingly, we note that only one OTU in the whole experiment (UMU_000011) (*Comamonadaceae*) was absent in the controls while reaching up to 5.3 % of relative abundance in tDOM + T mesocosms (Table 1). Furthermore, the same OTU (UMU_000011) was absent in the beginning of the experiment but was later recruited from what is frequently called the rare biosphere to tDOM_H_ + T mesocosms (Table 2). All other OTUs were always detected in both control and tDOM + T mesocosms (albeit sometimes in small numbers in one of the two, <0.01 % of relative abundance).

Within Alphaproteobacteria, we note that one *Roseobacter*-related OTU (UMU_000004) was equally abundant, between 10 and 16 % relative abundance, in both control and tDOM_H_ + T mesocosms (Fig. 2; Table 1). We also observed that members of the numerically abundant SAR11 clade (UMU_000067 and UMU_000003) responded positively, up to 13.8 % in relative abundance, in the tDOM_H_ + T mesocosms. Concomitantly, these SAR11 OTUs was lower, up to 6.5 %, in control mesocosms (Fig. 2; Table 1). Furthermore, an OTU (UMU_000026) closely related to *Candidatus* Spartobacterium Baltica1 (Herlemann et al. 2011, 2013) was predominantly abundant in control mesocosms and did not respond in tDOM_H_ + T mesocosms (Fig. 2).

A few OTUs increased substantially from being undetected to become abundant (>1 % of total abundance), as indicated above (UMU_000027, UMU_000011, UMU_000020). In addition to these OTUs, a *Desulfuromonadales* (Deltaprotebacteria) and a *Caldilinea* (Chloroflexi) also appeared in tDOM_H_ + T mesocosms after being undetected at first (Table 2).

## Discussion

In this mesocosm study with water from the northern Baltic Sea, we showed effects of increased tDOM and temperature in shaping bacterial community composition. These findings add important understanding of the bacterioplankton population dynamics in the Baltic Sea mesocosm experiment of Lefébure et al. (2013), who established that tDOM and temperature significantly affected bulk microbial activities. This is in agreement with studies in as diverse environments as the western Arctic, equatorial Pacific Ocean, and the Baltic Sea, all showing substantial combined effects of increased DOM and temperatures on bacterioplankton bulk activities (Kirchman and Rich 1997; Degerman et al. 2013) and overall general community structure (Degerman et al. 2013). Our findings further highlight that increased tDOM and temperatures promoted or suppressed a spectrum of individual populations (Fig. 2; Tables 1, 2). This study thus provides a comprehensive analysis of which bacterial populations may respond or not to future anthropogenic-induced shifts in environmental conditions.

### Differential response

Among the top 200 OTUs, around one-third showed relatively similar abundances in the tDOM_H_ + T and control mesocosms, suggesting that they were not affected by the induced changes in growth conditions—at least not within the time frame of the experiment. Another one-third of the OTUs increased in the tDOM_H_ + T mesocosms. Moreover, one-third of the OTUs were more abundant in control mesocosms, suggesting that they were negatively affected by increased temperature and tDOM. Overall, the analysis thus showed that 62 % of the top 200 OTUs in the current experiment were affected either positively or negatively by changes in environmental conditions. This suggests that persistent changes over periods from several months to years in temperatures and tDOM loading have the potential to cause profound changes in bacterioplankton community composition.

Several major bacterial groups that are abundant in the Baltic Sea were also abundant in our experiment. We find for example that Alphaproteobacteria were generally responsive (Fig. 2). Also Betaproteobacteria OTUs were mostly responsive, whereas Actinobacteria and phytoplankton were generally sensitive (Fig. 2). Still, within all major groups, there were both responsive and sensitive OTUs and even resistant ones. A possible reason for detecting equal increase or resistance among bacterial populations between control and tDOM_H_ + T mesocosms could potentially be the “bottle-effect”. Such effects are frequently seen in incubation experiments, especially among Gammaproteobacteria (e.g., Dinasquet et al. 2013). Nevertheless, despite possible “bottle-effects”, there were not only striking differences in terms of responsiveness, sensitivity, and resistance at low taxonomic resolution between major phylogenetic groups, but also differences within each phyla/class. Thus, we conclude that a majority of the responses observed were distinctive of the tDOM_H_ + T treatment as compared to the controls.

The following is an account of distinct distribution patterns for important individual populations. Betaproteobacterial OTUs like *Burkholderia* and *Comamonadaceae* were positively influenced by increased tDOM and temperature (Fig. 2; Tables 1, 2). In accordance, a study investigating the effects of continental runoff from the Iberian Peninsula on bacterioplankton showed a strong positive correlation between humic DOM and Betaproteobacteria (Teira et al. 2009). Although Betaproteobacteria are frequently found in small numbers in the Baltic Sea in general, specific members can reach up to several percent of the total community in the Baltic Sea (Herlemann et al. 2011, Lindh et al. 2015). In addition, the northern Baltic Sea contains on average more Betaproteobacteria than elsewhere in the Baltic Sea (Herlemann et al. 2011), possibly related to the lower salinity and/or higher levels of tDOM in this region. This suggests that the *Burkholderia* and BAL58 OTUs, in particular, and Betaproteobacteria, in general, may have an increased biogeochemical role in the cycling of carbon in the Baltic Sea and estuarine environments under future predicted climate change scenarios.

Alphaproteobacteria were generally stimulated by increased tDOM and temperatures, i.e., responsive, albeit some were found in both tDOM_H_ + T and control mesocosms, i.e., resistant. One particularly abundant Alphaproteobacteria was the resistant *Roseobacter* clade OTU UMU_000004 (Table 1). Members of the *Roseobacter* clade are often dominant (up to 25 % of total abundance) in marine surface waters around the globe (Newton et al. 2010) and relatives of this particular OTU have previously been found in the Baltic Sea (Sjöstedt et al. 2012). Many *Roseobacter* members contain metabolic features that allow them to be successful in various marine environments and are therefore of major importance for the cycling of carbon (Wagner-Dobler and Biebl 2006; Newton et al. 2010). Regarding members of the SAR11 clade bacteria, which are characterized as oligotrophs (Morris et al. 2002; Tripp 2013), it was surprising that two SAR11 OTUs were responsive to increased tDOM and temperatures, while a third was found primarily in the controls (Fig. 2; Table 1). In a previous Baltic Sea climate change experiment, a close relative of these SAR11 OTUs was predominant in higher temperatures (6 °C) but absent at lower temperatures (3 °C) (Lindh et al. 2013). Thus, although SAR11 clade bacteria are generally oligotrophs, it appears that different OTUs in this clade have a noticeable capacity to respond to changes in temperature and tDOM availability, considering that their abundance in seawater can have major implications for defining bacterioplankton community structure.

Actinobacteria are generally found in high abundance across the Baltic Sea, particularly in the northern basins (Bothnian Bay, Bothnian Sea) (Herlemann et al. 2011; Dupont et al. 2014, Lindh et al. 2015). Two important examples of sensitive actinobacterial OTUs, i.e., more abundant in control than in tDOM_H_ + T mesocosms, were members of the hgcI clade and the CL500-29 clade. The hgcI clade has previously not been described extensively among OTUs of the Baltic Sea, but relatives have been detected in high abundance in experiments with Baltic seawater (Sjöstedt et al. 2012). In lakes, members of the hgcI clade are often dominant components of the bacterioplankton, where they have a competitive advantage in waters with low DOC concentrations at low temperature (Glöckner et al. 2000). Still, bacteria in the hgcI clade remain poorly characterized and their functional traits in marine/brackish environments are unknown. The CL500-29 clade OTU found in high abundance in our control mesocosms has previously been found to be a generalist in terms of utilization of different carbon compounds in Baltic Sea microcosm experiments (Gomez-Consarnau et al. 2012). Thus, our results suggest a major decrease in the abundance of presently abundant actinobacterial populations in the northern Baltic Sea under predicted climate change scenarios.

Within Verrucomicrobia, we found 4 distinct OTUs related to the abundant but relatively unknown *Candidatus* Spartobacterium baltica that showed different responses in our mesocosms. This taxon is spatially widespread and abundant in the Baltic Sea (Herlemann et al. 2011), particularly during summer at times of cyanobacterial blooms and high temperatures (Andersson et al. 2009; Herlemann et al. 2011). This is likely due to the ability to utilize phytoplankton-derived high-molecular weight polysaccharides (Herlemann et al. 2013). In contrast, in our tDOM_H_ + T mesocosms, one of the Verrucomicrobial OTUs was outcompeted by other populations, which may suggest that it is less adapted to degrade and utilize terrigenous carbon-like humic substances. Still, it is important to note that other close relatives were either responsive or resistant to the control and tDOM_H_ + T conditions investigated here.

A similar distribution of differential responses was seen in the SAR86 clade, where one SAR86 OTU was responsive in tDOM_H_ + T mesocosms, and another was sensitive. Different members of this clade seem to have the capacity to degrade and utilize specific carbon compounds (Dupont et al. 2012), suggesting a possible differentiation into ecotypes. Thus, for several taxa, ecotype-level differentiation among closely related populations is important to consider when interpreting responses to changes in environmental conditions.

Bacteroidetes are often abundant in the Baltic Sea (Andersson et al. 2009; Herlemann et al. 2011, Lindh et al. 2015), and they are generally recognized for having an arsenal of enzymes to degrade phytoplankton-derived polysaccharides and peptides (Kirchman 2002; Fernandez-Gomez et al. 2013). Within the Bacteroidetes, there was substantial variation in response to the mesocosm conditions. For example, *Owenweeksia* OTUs were responsive, while members of the genus *Fluviicola* and the NS3a clade were sensitive to increased tDOM and temperature. Bacteroidetes often respond strongly to changes in growth conditions, either positively or negatively depending on which specific taxon/genus they belong to (Pinhassi et al. 2004; Andersson et al. 2009; Diez-Vives et al. 2014; von Scheibner et al. 2014). Substantial differences within Bacteroidetes in the number of glycoside hydrolases and peptidases are proposed to indicate a differentiation among taxa for distinct DOM utilization patterns (Fernandez-Gomez et al. 2013). This could account for parts of the variability among Bacteroidetes populations in degrading humic substances found in tDOM in our study.

In addition to major differences in the increase/decrease of OTUs, it was also curious to note that a few OTUs increased in relative abundance from being undetected at the onset of the experiment (Table 2). In particular, opportunistic populations, such as *Comamonadaceae* (Betaproteobacteria) and *Desulfuromonadales* (Deltaprotebacteria) OTUs, increased substantially in tDOM_H_ + T mesocosms. Rare, or initially undetected OTUs that becomes abundant also occurs in situ in the marine environment and has been observed in experimental incubations following environmental perturbations, emphasizing the role of the rare biosphere in responding to change in environmental conditions (Campbell et al. 2011; Lennon and Jones 2011; Sjöstedt et al. 2012; Alonso-Saez et al. 2014). For example, change in salinity promoted previously rare or undetected OTUs in chemostat transplants between Skagerrak seawater and Baltic Sea water (Sjöstedt et al. 2012).

It is also important to note that the observed changes among bacterial populations in our experiment are the result of adaptation in a closed system. The distribution of bacterial populations in the natural marine environment is limited by few physical barriers and, in the perspective of climate change, dispersal is likely an important factor for bacterioplankton responses to environmental change. Nevertheless, our observations highlight substantial effects of climate change-induced shifts in the local environmental conditions for regulating bacterioplankton community composition

## Conclusions

The observed shifts in bacterial community composition that we report here link to concomitant changes in community metabolism as reported by Lefébure et al. (2013). Notably, Lefébure et al. (2013) showed that bacterial production increased substantially in tDOM_H_ + T compared to control mesocosms, indicating that the response of community metabolism under the manipulated environmental conditions could affect ecosystem functioning in brackish seawater. The current study thus contributes detailed insights into how the response in community metabolism was linked to the increase/decrease in the abundance of specific bacterial populations. Bacterioplankton composition is increasingly viewed as a factor that contributes to controlling ecosystem functioning (Bell et al. 2005; Comte and Del Giorgio 2011). Both adaptation and replacement of OTUs have been observed in other aquatic systems (Langenheder et al. 2005; Comte and Del Giorgio 2011) emphasizing the presence of both generalist and specialist populations. Altogether, these findings suggest that environmental disturbances induced by anthropogenic activities, such as increased precipitation and sea surface temperatures, are liable to cause alterations in microbially mediated ecosystem functions and carbon fluxes, ultimately promoting heterotrophy in brackish seawater systems.
